# Supplementary material for: Functional and genetic determinants of mutation rate variability in regulatory elements of cancer genomes
Source: Genome Biol. 2021 May 3;22:133. doi: 10.1186/s13059-021-02318-x (PMC8091793; doi:10.1186/s13059-021-02318-x)
Supplement: Supplementary file 3 — Additional file 3. Review history. [file 13059_2021_2318_MOESM3_ESM.docx]

Review History

**First round of review**

**Reviewer 1**

**Are you able to assess all statistics in the manuscript, including the appropriateness of statistical tests used?**

No, I do not feel adequately qualified to assess the statistics.

**Comments to author:**

Major points

1. p7 " To obtain a conservative analysis, we excluded a small fraction of tumors as outliers (31 or 1.3%) where even single-sample RM2 analysis revealed highly significant differences in local mutation rates in any of the three classes of sites (FDR < 0.001) (Supplementary Figure 3B)". What explains the outlier behaviour of these samples with RM2 analysis? Also, why does Supp Fig 3B suggest that there are 37 outliers not 31?

2. p7-9 Although the RM2 model is novel it is highly questionable that the pancancer patterns of mutation enrichment at CTCF, TSS and ATAC-seq peak sites are novel, or unexpected given the prior literature. This is tacitly conceded by the authors as they seem to view these sites as positive controls (p10).

3. p9 Where the authors' observations do contradict the prior literature (eg "Our findings contrast an earlier report that indicated broadly decreased mutation rates at chromatin-accessible regulatory elements derived from cell lines") they make no effort to explain this. I think a likely explanation is that the authors have used different annotations from prior studies, which should be explained in the text.

4. The authors have based their mutation rate analysis (p7-9) on genomic annotations derived from many different cell types (CTCF peaks observed in >1 ENCODE cell line, the pan-cancer ATAC-seq peaks from TCGA and all protein-coding TSSs from Ensembl) and many of these sites will not be functional in any given cancer type. For example the TCGA ATAC-seq study found that around half of all pan-cancer distal ATAC-seq sites were specific to particular cancer types, so it is likely that many of these sites are not functional in the cancer types studied by the authors. It therefore seems difficult to interpret the results in figures 1 and 2 which seem to assume that all sites are functional in all cancers. How do we distinguish real differences in mutation rate between cancer types from differences arising simply from variation between types in the usage of TSSs, CTCF and ATAC-seq sites?

5. p13 The authors present evidence for elevated mutation rates at TSSs as the result of a " transcription-dependent mutational process" but then go on to suggest that "some more frequent non-coding mutations at individual promoters are functional and may contribute to cancer driver mechanisms". What is the evidence that any of the noncoding mutations appearing at promoters affect gene function? Also, what is the evidence that such mutations drive tumourigenesis?

6. p13-14 The elevation of mutation rates at constitutively bound CTCF sites and chromatin loop anchors are not novel findings and have been observed previously in ICGC cancer mutation data (eg Pinoli et al, 2020, PLoS One 15(1): e0227180; Kaiser and Semple, 2018, Genome Biol 19:101). At the very least these studies should be referenced.

7. p17-19 The authors detect associations between local mutation rates and driver mutations or CNA and describe "examples of potential genetic factors contributing to local mutagenesis". The text from p17 to p19 contains many similar statements implying a causal relationship between a particular driver mutation or CNA and elevated mutation rates at particular sites. It would appear that there is no evidence for such a causal relationship, such that these CNAs/drivers "contribute to" or "drive" mutation rates. There seems to be no reason to suggest that these associations or correlations reflect causation and the text should be amended to reflect this.

Minor points

- The ms needs to be checked for typos - which are present here and there eg in the second sentence: " confer cells select proliferative advantages".

**Reviewer 2**

**Are you able to assess all statistics in the manuscript, including the appropriateness of statistical tests used?**

Yes, and I have assessed the statistics in my report.

**Comments to author:**

The manuscript by Lee et al. presents an interesting statistical framework to identify local mutational rate variability in regulatory regions and applied this to a set of CTCF binding sites, TSS and open chromatin regions. At first, the method present here is novel compared to the existing methods, which were focused on the identification of specific genomic loci that have an unexpected mutation rate. Secondly, the application of this method revealed some interesting findings including the identification of specific mutation types and signatures enriched in the regulatory regions, and the correlation of specific cancer driver alterations associated with the increased mutation rate variability in the regulatory regions. Overall, the manuscript is well written and the results are discussed in detail. The following are some comments suggested for improvements.

a) While defining the genomic regions in RM2, it is not clear whether the control regions (i.e., flanking regions ) is devoid of any overlapping regulatory regions? For example, if the two CTCF binding sites are adjacent to each other, one of them could well be within the flanking region limit of others. This might potentially bias the expected mutation rate calculation.

b) Given that the method quantifies the local or regional mutation rate variation (by taking adjacent flanking region as control), it is not clear what is the importance of this megabase-scale covariates parameter (MbpRate)? If we remove this parameter, how much does it affect the estimates?

c) Lines 155-162, the reported fold-change for the observed versus expected mutation rate in CTCF binding seems to be much lower (<2), as compared to the signal strength previously shown in the studies such as Katainen et al., 2015 https://doi.org/10.1038/ng.3335 and Poulos et al., 2016 http://dx.doi.org/10.1016/j.celrep.2016.11.055), where they have taken the motif centre as mid-point instead of chip-seq peak signal.

d) Lines 213-214: "Our findings contrast an earlier report that indicated broadly decreased mutation rates at chromatin-accessible regulatory elements derived from cell lines". This seems to be an overstatement because overall the enrichment of SNV mutation rate in slightly higher in the open chromatin regions in certain tumor types (as shown in Figure 1A, also the fold change in much lower compared to the CTCF or TSS analysis). However, this could be an artefact due to the consideration of pan-cancer ATAC-seq peaks (instead of matched tumor type-specific peaks). Also, the enrichment found in Skin-Melanoma (for example) could be driven by the TSS or TFBS sites that were covered within the ATAC-seq peaks.

e) Lines 281-285: "Furthermore, the significant enrichment of TSS-specific mutations in genes of cancer-related processes suggests that some more frequent non-coding mutations at individual promoters are functional and may contribute to cancer driver mechanisms and tumor heterogeneity by altering gene-regulatory circuits in molecular pathways and interaction networks".

Given that most of the genes/pathways identified were from skin-melanoma. The authors may need be more specific here, because the previous study by Fredriksson et al., 2017 (https://doi.org/10.1371/journal.pgen.1006773) has shown that the recurrent mutation in the promoter regions was not associated with the differential expression of the nearby genes in melanomas.

f) Lines 332-333: "Driver mutations in BRAF in melanoma were associated with an increased mutation rate at CTCF binding sites"

This is interesting. is this association specific to CTCF binding sites or other TFBSs also (which were also previously shown to have increased mutation rate)? And, what is the association with the increased mutation rate at TSS centred analysis?

**Authors Response**

**Point-by-point responses to the reviewers’ comments:**

Reviewer #1:

Major points

1. p7 " To obtain a conservative analysis, we excluded a small fraction of tumors as outliers (31 or 1.3%) where even single-sample RM2 analysis revealed highly significant differences in local mutation rates in any of the three classes of sites (FDR < 0.001) (Supplementary Figure 3B)". What explains the outlier behaviour of these samples with RM2 analysis? Also, why does Supp Fig 3B suggest that there are 37 outliers not 31?

*Overall, the outlier tumors have a higher mutation burden (mean 50,656 vs. 9,296 SNVs/indels per tumor). We have added this note to our manuscript. However, the tumors are not hypermutated according to our definition (>90,000 mutations). Hypermutated tumors were removed prior to this analysis.*

*We would prefer not to speculate about the characteristics of the few outlier tumors since the set is small and heterogeneous: there are nine cancer types and some tumors are detected as outliers in one type of site (i.e., TSS, CTCF, or open-chromatin) while others are outliers in multiple site types. Conclusive statements would require a great deal of analysis separate from the theme of this paper. We would gladly study this in a future project.*

*We excluded these tumors because we found that they had a disproportionate effect on our localised mutation frequency analysis throughout the study. By doing so, we generated a far more conservative set of results and captured patterns shared a¬cross multiple tumors.*

*The number of outlier samples is now 33, while the current figure (Supplementary Figure 1B) displays 38 points in total. The number of colored points in the figure (38) is correct: it is larger than the number of unique outlier tumors since some tumors were identified as outliers in several types of sites. We noted this in the figure caption.*

*That said, a conceptually similar analysis to associate genetic characteristics with localised mutation frequencies is presented in Figure 5 (previously Figure 4).*

2. p7-9 Although the RM2 model is novel it is highly questionable that the pancancer patterns of mutation enrichment at CTCF, TSS and ATAC-seq peak sites are novel, or unexpected given the prior literature. This is tacitly conceded by the authors as they seem to view these sites as positive controls (p10).

*We would like to thank the reviewer for highlighting the novelty of our method. We have now created a new Figure 1 to describe our method and its performance (these materials were previously shown in Supplementary Figures 1-2). Figures 2-5 have been renumbered.*

*We agree with the reviewer that elevated mutation frequency of certain regulatory elements in cancer genomes is known (i.e., in CTCF sites). We have mentioned this in the Introduction and Results sections and have since included additional references. We believe that the confirmed results are important to include because these show the validity of our model and provide a gateway to the rest of the study where we perform novel in-depth analyses.*

*We are confident that our analysis builds on this previous knowledge and believe that the following aspects of our study are novel and innovative:*

*1.	Pan-cancer analysis of >2,400 whole cancer genomes and 25 cancer types in a high-quality uniformly processed dataset. Earlier studies have analyzed fewer genomes and fewer cancer types. As a result, even when reporting previously discovered results (such as T>G substitutions at CTCF sites in GI cancers), we are showcasing the pattern across unexplored cancer types and allow a side-by-side comparison.*

*2.	Analysis of open-chromatin elements from cancer tissues (ATAC-seq) and matching normal tissues (Epigenomics Roadmap) using genomic profiles of matching tumor types, where possible. In contrast, previous studies have used open-chromatin profiles derived from cell lines. In the updated manuscript, we use specific sets of open-chromatin sites for every tumor type (comment (4) below). In the updated analysis, the mutation enrichment of open-chromatin sites appears stronger.*

*3.	Association of elevated mutation frequencies with mRNA abundance (in several cancer types other than melanoma). This analysis uses matched mRNA and WGS data from the same tumor samples in multiple cancer types.*

*4.	Observations of elevated TSS mutation frequencies in specific pathways and processes, including cancer-associated pathways.*

*5.	The observation that the strongest mutation enrichments occur at CTCF binding sites constitutively active in 67 cell types and also match the core CTCF DNA-binding motif. This extends previous work by comparing levels of shared activity in a large and diverse set of CTCF binding sites (representing ~160,000 unique sites in 70 cell lines). The PCAWG cohort of 25 cancer types with whole-genome sequencing adds novelty.*

*6.	Mutational signatures associating with high-activity transcription start sites (i.e., those with high mRNA abundance of target genes) and high-activity CTCF sites (i.e., those active in most or all cell types). The high-activity bins of sites show enrichments of characteristic mutational signatures of the respective cancer type. This new analysis has been added as Supplementary Figure 3.*

*7.	Genetic alterations associating with elevated mutation frequencies at gene-regulatory and chromatin architectural elements. For example, BRAF driver mutations associating with elevated CTCF binding site mutations in melanoma, ARID1A mutations associating with elevated TSS-specific mutation frequencies in pancreatic cancer, and recurrent amplifications of 17q23.1 associating with CTCF binding site mutations in pancreatic and breast cancer (added in this revision).*

*We have now emphasized these aspects of novelty and conceptual advances in the Results and Discussion sections and added more references where appropriate.*

3. p9 Where the authors' observations do contradict the prior literature (eg "Our findings contrast an earlier report that indicated broadly decreased mutation rates at chromatin-accessible regulatory elements derived from cell lines") they make no effort to explain this. I think a likely explanation is that the authors have used different annotations from prior studies, which should be explained in the text.

*Thank you, this is a great point. One of the advances of our study is that the localised mutation frequency analysis considers tissue-specific open-chromatin maps (i.e., ATAC-seq or DNA-seq profiles) derived from primary tumors of matching cancer types, instead of open-chromatin profiles of common cell lines or normal tissues used in earlier studies. We have also used a much larger set of whole cancer genomes of the PCAWG project that have been processed consistently using state-of-the-art pipelines. In this revision, we have updated the analysis of open-chromatin sites to use tissue-specific profiles as discussed in the next comment.*

*We have added a few sentences to the Discussion section to emphasize the novelty of our study with respect to the cancer datasets we used.*

4. The authors have based their mutation rate analysis (p7-9) on genomic annotations derived from many different cell types (CTCF peaks observed in >1 ENCODE cell line, the pan-cancer ATAC-seq peaks from TCGA and all protein-coding TSSs from Ensembl) and many of these sites will not be functional in any given cancer type. For example the TCGA ATAC-seq study found that around half of all pan-cancer distal ATAC-seq sites were specific to particular cancer types, so it is likely that many of these sites are not functional in the cancer types studied by the authors. It therefore seems difficult to interpret the results in figures 1 and 2 which seem to assume that all sites are functional in all cancers. How do we distinguish real differences in mutation rate between cancer types from differences arising simply from variation between types in the usage of TSSs, CTCF and ATAC-seq sites?

*Thank you, this is a great comment. We have revised the analysis of open-chromatin sites by including tissue-specific sites. This update improved our study since enrichments in tissue-specific open-chromatin sites appear stronger than in the pan-cancer sites. For the other two site classes (TSS, CTCF binding sites), our analyses already account for tissue-specific activity.*

*First, for open-chromatin sites, we have included tissue-specific sets of open-chromatin sites for all cancer types (instead of the pan-cancer set of sites used in the original manuscript). The analysis is shown in Figure 2A and elsewhere in the manuscript. This was also mentioned by Reviewer 2 (comment (d)). We matched 17/25 cancer types of the PCAWG datasets to ATAC-seq peaks of the same cancer types in the TCGA dataset (Corces et al. 2018). For 8/25 cancer types, we used DNAse-seq profiles of the closest relevant normal tissues from Epigenomics Roadmap. For the pan-cancer cohort, we continued to use pan-cancer ATAC-seq profiles from TCGA. This information is available in Supplementary Table 1A. To better deconvolute the open-chromatin analysis, we also removed the open-chromatin sites that occurred at TSSs (Reviewer 2, (d)). Therefore, the analysis of open-chromatin sites now better reflects the sites active in different cancer types. The updated analysis remains largely consistent with our original findings, however the mutation enrichments at open-chromatin sites now appear stronger.*

*Second, for TSSs, we already consider their tissue-specific activity in the relevant cancer types (Figure 3; previously Figure 2). We group the TSSs by mRNA abundance of target genes in the same tumor samples. We use mRNA abundance (RNA-seq) data of the PCAWG project that also provides the mutation data (~50% of tumors have RNA-seq data in PCAWG). For example, we show that TSSs of transcriptionally silent genes (i.e., the TSSs not used in a given cancer type) have little or no relative increase in mutation frequencies. In contrast, highly expressed genes (i.e., highly active TSSs in a given cancer type) have elevated TSS-specific mutation frequencies in that cancer type. We have revised the Results section to clarify this better.*

*Third, for CTCF binding sites, we already consider site activity across multiple cell types as a proxy of activity (Figure 4, previously Figure 3). We assumed that the more consistently-bound is a given CTCF binding site across the 70 cell types, the more likely the site is functional in a given cancer type. In Figure 4, we show that the largest mutation frequency increases affect the CTCF binding sites that are active in in nearly all cell types while the sites with lower conservation are not mutated. We use this estimate of functionality because CTCF DNA-binding profiles of primary tumors are not available for various cancer types. Cell line profiles are available, however, matching all cancer types with appropriate cell lines comprehensively was not possible. We have revised the Results section to clarify this in the manuscript.*

*Lastly, to address the comment on tissue-specific activity of CTCF, we analysed CTCF binding sites in matching cell lines for two cancer types (Supplementary Figure 4). We confirmed the observation of specific mutation enrichments at constitutively active CTCF binding sites.*

*-	Breast cancer mutations (the PCAWG Breast-AdenoCa cohort) analysed in the CTCF binding sites of the breast cancer cell line MCF-7 (ENCODE),*

*-	Liver cancer mutations (the PCAWG Liver-HCC cohort) analysed in the CTCF binding sites of the liver cancer cell line HepG2 (also ENCODE).*

5. p13 The authors present evidence for elevated mutation rates at TSSs as the result of a " transcription-dependent mutational process" but then go on to suggest that "some more frequent non-coding mutations at individual promoters are functional and may contribute to cancer driver mechanisms". What is the evidence that any of the noncoding mutations appearing at promoters affect gene function? Also, what is the evidence that such mutations drive tumorigenesis?

*Thanks for highlighting this speculative sentence. In response to this comment and another one (Reviewer 2, (e)), we removed the sentence from the Results section. Deciphering the interplay of positive selection and neutral mutational processes remains a challenge. Our current work helps understand localised mutational processes and their functional and genetic covariates. This can inform the construction of better computational models to identify coding and non-coding driver mutations. We extended the relevant paragraph in the Discussion section where such statements are more appropriate.*

*In our opinion, analysing non-coding driver mutations remains out of scope of this study. In particular, the RM2 method is not appropriate for identifying positive selection in single genomic sites as it is designed for larger numbers of sites (thousands and more). The topic of non-coding driver discovery is thoroughly addressed elsewhere, and recent studies report candidate driver mutations in non-coding regulatory elements using different techniques. For example, the initial sentence cited a recent study from the PCAWG project that reported the convergence of non-coding low-frequency candidate driver mutations in the regulatory elements of cancer-related pathways and protein interaction networks [Reyna et al, Nature Comms 2020; PMID:32024854].*

6. p13-14 The elevation of mutation rates at constitutively bound CTCF sites and chromatin loop anchors are not novel findings and have been observed previously in ICGC cancer mutation data (eg Pinoli et al, 2020, PLoS One 15(1): e0227180; Kaiser and Semple, 2018, Genome Biol 19:101). At the very least these studies should be referenced.

*Thanks for the comment. We have included both references and extended the text on how we built upon previous studies. Please also see our response to a related comment below (Reviewer 2, (c)).*

*We believe that our analysis of mutation frequencies at constitutive CTCF binding sites (Figure 4, previously Figure 3) extends these earlier studies. We consider CTCF ChIP-seq profiles of 70 cell lines to quantify the conservation of CTCF binding (compared to, for example, the analysis of CTCF motifs at the loop anchors of seven cell lines in Kaiser and Semple, 2018).*

*This larger number of cell lines allowed us to assess mutation frequencies at different levels of CTCF binding site activity. We determined the top 20% of constitutively active CTCF binding sites (median conservation in 67 of 70 cell lines) where the mutational enrichments were the strongest. All other bins showed no enrichment of mutations, thus our extended and scaled classification is more comprehensive. For example, bin 4 that contains CTCF sites with high binding conservation (27 of 70 cell lines, median) showed no significant mutation enrichment.*

*Our results span 25 well-powered cancer types and the pan-cancer cohort of a large, uniformly processed dataset. In conjunction with the levels of site activity, we show that this mutation enrichment of CTCF binding sites is shared across eleven cancer types. We have improved this analysis and included a new panel to show the fold-change and distribution of mutations in the CTCF sites of different bins (Figure 4D, copied below).*

*We further addressed this comment by grouping all the CTCF binding sites by the presence or the absence of CTCF DNA-binding motifs in the sites (Figure 4E,F; Supplementary Figure 4). We analysed ten bins of sites in total: five bins with motifs, and five bins without motifs, all grouped by their level of binding conservation in the 70 cell lines. Strikingly, we found that the constitutive CTCF sites with motifs (bin 5) were the focus of the mutational enrichment, while the constitutive CTCF sites without motifs (also bin 5) had no or little enrichment of mutations. We also confirmed this observation in an analysis of cell line specific CTCF sites for two cancer types with matching mutations (breast and liver cancer). We discuss these results in the response to comment (c) of Reviewer 2.*

7. p17-19 The authors detect associations between local mutation rates and driver mutations or CNA and describe "examples of potential genetic factors contributing to local mutagenesis". The text from p17 to p19 contains many similar statements implying a causal relationship between a particular driver mutation or CNA and elevated mutation rates at particular sites. It would appear that there is no evidence for such a causal relationship, such that these CNAs/drivers "contribute to" or "drive" mutation rates. There seems to be no reason to suggest that these associations or correlations reflect causation and the text should be amended to reflect this.

*Thanks to the reviewer for this comment. We agree and have already deliberately avoided the word “causal” since our analysis is associative. A careful set of experiments in cell lines and animal models would be needed to imply causality. We have revised this paragraph and explicitly state that we cannot identify causal relations and the study remains associative.*

*With that said, we cite studies where functional experiments were performed to support our observations. For the ARID1A association with TSS-specific mutation rates (added in the revision), we cite several studies documenting the role of this tumor suppressor gene as a genomic gatekeeper in controlling chromosomal stability and DNA repair [e.g., Dykhuizen et al. Nature 2013, PMID:23698369]. For BRAF mutations in melanoma associating with localised mutagenesis at CTCF sites, we cite a study that expressed the BRAF-V600E mutant gene in epithelial cell lines and observed increased levels of DNA double-strand breaks and reactive oxygen species [Sheu et al. Am J. Pathol. 2013, PMID:22227015]. The earlier studies lend confidence to our analysis and support our findings, so we believe a certain amount of forward-looking speculative language is justified.*

Minor points

- The ms needs to be checked for typos - which are present here and there eg in the second sentence: " confer cells select proliferative advantages".

*Thank you. We have reviewed the text in detail and hopefully fixed all remaining typos.*

Reviewer #2:

The manuscript by Lee et al. presents an interesting statistical framework to identify local mutational rate variability in regulatory regions and applied this to a set of CTCF binding sites, TSS and open chromatin regions. At first, the method present here is novel compared to the existing methods, which were focused on the identification of specific genomic loci that have an unexpected mutation rate. Secondly, the application of this method revealed some interesting findings including the identification of specific mutation types and signatures enriched in the regulatory regions, and the correlation of specific cancer driver alterations associated with the increased mutation rate variability in the regulatory regions. Overall, the manuscript is well written and the results are discussed in detail. The following are some comments suggested for improvements.

*We would like to thank the reviewer for the positive comments and great recommendations for improvements. Following the positive remarks regarding our method by both reviewers, we have created a new Figure 1 that provides an overview of the method and benchmarking analyses (previously shown in Supplementary Figures 1-2). Consequently, the Figures 2-5 have been renumbered.*

a) While defining the genomic regions in RM2, it is not clear whether the control regions (i.e., flanking regions) is devoid of any overlapping regulatory regions? For example, if the two CTCF binding sites are adjacent to each other, one of them could well be within the flanking region limit of others. This might potentially bias the expected mutation rate calculation.

*Thank you for a great comment. We have now updated our method such that every nucleotide and every mutation is either accounted for a site or a flanking sequence but is never accounted twice. This is achieved by merging the related genomic regions and assigning the regions and the mutations into either of the two classes (site vs. flank). In our previous version, we had handled the issue by only removing duplicate instances of mutations (i.e., those affecting both sites and flanking sequences) that led to an overall underestimate of mutation frequencies both in the sites and the flanking regions. We revised the Methods section to reflect these changes.*

*We repeated our analyses several times and carefully inspected the results. The vast majority of our findings remain consistent with the initial manuscript. Two major changes occurred:*

*-	We detect stronger mutation enrichments in open-chromatin regions in most cancer cohorts (Figure 2A), likely because our updated analysis considers tissue- and cancer-specific open-chromatin sites rather than pan-cancer sites. See Comment (4) of Reviewer 1 and Comment (d) below.*

*-	The section on genetic cofactors of site-specific mutation enrichments was updated (Figure 5). We now highlight ARID1A driver mutations associating with elevated mutation frequency at TSSs in pancreatic cancer. Two results highlighted previously (RAD21 at 8q23; SHDA at 5p15.33) were no longer detected as significant so we deemphasized those. BRAF mutations associating with CTCF binding site mutations in melanoma remain consistent with the previous manuscript.*

b) Given that the method quantifies the local or regional mutation rate variation (by taking adjacent flanking region as control), it is not clear what is the importance of this megabase-scale covariates parameter (MbpRate)? If we remove this parameter, how much does it affect the estimates?

*We now compared the models of localised mutation frequencies with and without the MbpRate covariate and evaluated the resulting P-values in true mutations and simulated mutations in multiple cancer types (Figure 1C; copied below). Our results indicate the importance of the covariate in the model.*

*Theoretically, we included this covariate because the sites are spread throughout the genome and are subject to megabase-scale variation in mutation frequencies (Lawrence et al, Nature 2013, PMID:23770567). Nearby sites that are separated on the order of hundreds to thousands of nucleotides have similar megabase-level mutational frequencies and will typically fall into the same or adjacent MbpRate mutation bins, whereas bins of distal sites may drastically differ. Therefore, we hypothesized that this covariate would help control some of these broader-scale differences that would confound model predictions of mutation counts at the local scale.*

*Statistically, we found that the inclusion of the MbpRate covariate increased the significance of findings in the true mutation dataset (i.e., the red dots located below the diagonal line, left panel). This suggests an increased sensitivity of the model that uses the megabase-scale covariate. In contrast, the use of MbpRate parameter in the model assigned lower significance to results in the simulated mutation dataset (i.e., the gray dots located above the diagonal line; right panel), indicating that the covariate also protects against potential false-positive results. Also, the MbpRate coefficient itself is always highly significant in the regression models according to ANOVA analyses (data not shown).*

*In summary, evaluating the localised mutation frequency differences across multiple bins of sites allows us to better evaluate the variability of mutation frequency between sites and flanking regions in individual trinucleotide contexts and it may lead to better-powered models as a result. We have discussed this analysis in our Results section.*

c) Lines 155-162, the reported fold-change for the observed versus expected mutation rate in CTCF binding seems to be much lower (<2), as compared to the signal strength previously shown in the studies such as Katainen et al., 2015 https://doi.org/10.1038/ng.3335 and Poulos et al., 2016 http://dx.doi.org/10.1016/j.celrep.2016.11.055), where they have taken the motif centre as mid-point instead of chip-seq peak signal.

*Thanks, this is a good point. We addressed the comment with additional analyses described below and improved the description of results in the manuscript.*

*We added an analysis where the conservation-based grouping of CTCF binding sites was combined with the presence or absence of the consensus core CTCF DNA-binding motif (FDR < 0.01; the FIMO method). This approach revealed clearly elevated fold-changes in the most frequently mutated groups of sites (Figure 4E,F copied below; Supplementary Figure 4). We found a striking mutation enrichment in constitutively bound CTCF sites that include a core motif, compared to the other groups of sites where the CTCF binding was not constitutively active, or where the DNA motif was absent. We confirmed that core CTCF DNA motifs were overall enriched in the 5th constitutively active bin of CTCF binding sites (Figure 4B).*

*This motif- and conservation-based mutation enrichment was confirmed in another analysis where we considered the CTCF binding sites of relevant cancer cell lines to analyse mutations of matched cancer types (Supplementary Figure 4). Liver cancer mutations were analysed in the CTCF binding sites of the HepG2 liver cancer cell line, and breast cancer mutations were analysed in the CTCF binding sites of the MCF-7 breast cancer cell line, and the conservation of those sites was evaluated across the remaining cell lines in ENCODE.*

*These analyses better describe the CTCF-related mutational processes and highlight the functionally annotated subsets of sites where the fold-change values are higher.*

*There are a few additional explanations for these lower fold-changes we observed.*

*•	First, we used the ChIP peak centre as the site midpoint rather than the motif midpoint. We opted for this more generalizable approach since not all experimentally captured CTCF binding sites closely match the consensus core motif, and not all transcription factors have identifiable DNA-binding motifs.*

*•	Second, we compare larger sites (50 bps) than just the core motif (19 bps), therefore less-mutated nucleotides around the site midpoint contribute to lower fold-changes. This provides leeway for cases where the motif and peak midpoints are not perfectly aligned.*

*•	Third, we use a large collection of experimentally-derived CTCF binding sites of 70 cell lines from ENCODE. These represent at least a certain fraction of non-specific CTCF binding events with a sub-optimal DNA motif or lack a consensus core motif overall.*

*•	Fourth, in this study we cautiously removed a subset of tumor samples that were so strongly hypermutated in the context of CTCF, TSS and/or open chromatin sites that they alone could capture significant results. Consequently, we used a more conservative approach to ensure that the discovered associations were shared in many genomes.*

d) Lines 213-214: "Our findings contrast an earlier report that indicated broadly decreased mutation rates at chromatin-accessible regulatory elements derived from cell lines". This seems to be an overstatement because overall the enrichment of SNV mutation rate in slightly higher in the open chromatin regions in certain tumor types (as shown in Figure 1A, also the fold change in much lower compared to the CTCF or TSS analysis). However, this could be an artefact due to the consideration of pan-cancer ATAC-seq peaks (instead of matched tumor type-specific peaks). Also, the enrichment found in Skin-Melanoma (for example) could be driven by the TSS or TFBS sites that were covered within the ATAC-seq peaks.

*Thanks for a great comment. In this sentence, we wanted to say that our study found an overall elevation of mutation frequencies in open-chromatin regions. In contrast, this previous report (Polak et al. Nat Biotech 2014) found a reduction of mutation frequencies at open-chromatin regions. Potential explanations are that previous studies did not include cancer-specific open-chromatin regions, studied fewer genomes and did not account for megabase-scale mutation frequencies as covariates. Also, Polak et al. used a broader control region around sites for comparison that could contribute to this discrepancy. We added a paragraph in the Discussion section to discuss this difference of our study and the previous report, emphasizing the importance of using tissue-specific open-chromatin sites.*

*To address this comment, we have updated this analysis by selecting tissue-specific open-chromatin sites for the individual cancer types (see Reviewer 1 comment (4)). We also removed the open-chromatin sites in each tissue that overlapped transcription start sites (TSS) and also CTCF binding sites. These steps were taken to avoid potential confounding effects, as suggested by the reviewer.*

*Our updated results indicate broad increases of mutation frequencies in open-chromatin sites. The significance values and fold-changes are more pronounced after including tissue-specific sites (Figure 2A,D; previously Figure 1). This is apparent in all mutations combined, various mutational signatures, and when grouping the sites by target gene expression (Figure 3A,B, previously Figure 2). The improved signal underlines the importance of using matching genomic and epigenomic information to study the interactions of chromatin state and mutational processes. We have better emphasized this result in the manuscript.*

e) Lines 281-285: "Furthermore, the significant enrichment of TSS-specific mutations in genes of cancer-related processes suggests that some more frequent non-coding mutations at individual promoters are functional and may contribute to cancer driver mechanisms and tumor heterogeneity by altering gene-regulatory circuits in molecular pathways and interaction networks". Given that most of the genes/pathways identified were from skin-melanoma. The authors may need be more specific here, because the previous study by Fredriksson et al., 2017 (https://doi.org/10.1371/journal.pgen.1006773) has shown that the recurrent mutation in the promoter regions was not associated with the differential expression of the nearby genes in melanomas.

*We have revised this sentence since we agree that it would appear speculative in this Results section (see also Reviewer 1; (5)). Non-coding driver analysis is not a focus of this study since RM2 was not designed to detect positive selection in single sites. However, the effects of positive selection (i.e., reflecting non-coding driver mutations) and localised mutational processes (i.e., generating passenger mutations) are intertwined and dissecting the two effects conclusively for any given site is difficult and goes beyond this study. The initial sentence had attempted to tie our pathway-based enrichment signals with previous literature, as it cited a recent study of the PCAWG consortium that identified non-coding promoter mutations and associated mRNA changes using pathway and network analysis approaches (Reyna et al, Nature Communications 2020; https://pubmed.ncbi.nlm.nih.gov/32024854/).*

*Instead of the sentence above, we added a paragraph in the Discussion section about the interplay of local mutational processes and their potential functional impact and putative non-coding driver mutations.*

f) Lines 332-333: "Driver mutations in BRAF in melanoma were associated with an increased mutation rate at CTCF binding sites". This is interesting. is this association specific to CTCF binding sites or other TFBSs also (which were also previously shown to have increased mutation rate)? And, what is the association with the increased mutation rate at TSS centred analysis?

*Thanks for a great comment. We added a control analysis by comparing mutations at TSSs and open-chromatin sites in BRAF-wildtype and BRAF-mutant melanoma genomes (Supplementary Figure 6). Neither class of sites shows a significant interaction with BRAF mutations as the statistical interactions from the joint model are not significant (P > 0.05), FDR values of BRAF-wildtype and BRAF-mutant groups are both significant when analysed separately, and the fold-changes (sites vs. flanks) are relatively similar for both subgroups of tumors (1.19 vs. 1.15; 0.99 vs. 1.03). This suggests that the association of BRAF mutations and localised mutations is more specific to CTCF binding sites than the other classes of sites we studied.*

**Second round of review**

**Reviewer 1**

The manuscript is much improved and I have no further comments.

**Reviewer 2**

The authors have adequately addressed my comments.
